# The Dark Side of STING Signaling In Niemann‐Pick Disease Type C And Other Neurodegenerative Diseases

**DOI:** 10.1002/alz.084589

**Published:** 2025-01-03

**Authors:** Ting‐Ting Chu

**Affiliations:** ^1^ Shenzhen Bay Laboratory, Shenzhen, Guandong China

## Abstract

**Background:**

The classic mode of STING activation is through binding the cyclic dinucleotide 2′3′‐cyclic GMP–AMP (cGAMP), produced by the DNA sensor cyclic GMP–AMP synthase (cGAS), which is important for the innate immune response to microbial infection and autoimmune disease. Modes of STING activation that are independent of cGAS are much less well understood. We wanted to explore the interactome of STING on the organelles during its trafficking route and to understand the regulatory network of STING signaling.

**Method:**

To achieve this, we conducted an unbiased spatiotemporally resolved proximity labeling screen, followed by quantitative proteomics. This led us to discover a novel regulator of STING, NPC1, which is a lysosomal membrane protein that plays a role in cholesterol efflux. Mutations in NPC1 can cause Niemann Pick disease Type C, a neurodegenerative disease. Genetic deletion of *Sting1* (the gene that encodes STING) or *Irf3*, but not that of *Cgas*, significantly reduced the activation of microglia and relieved the loss of Purkinje neurons in the cerebellum of *Npc1^−/−^* mice, leading to improved motor function.

**Result:**

We found that NPC1 interacts with STING and recruits it to the lysosome for degradation in both human and mouse cells. Our findings also revealed that knockout of *Npc1* ‘primes’STING signaling by physically linking or ‘tethering’ STING to SREBP2 trafficking. Loss of NPC1 protein also ‘boosts’ STING signaling by blocking lysosomal degradation. Both priming and boosting of STING signaling are required for severe neurological disease in the *Npc1^−/−^* mouse

**Conclusion:**

Both priming and boosting of STING signaling are required for severe neurological disease in the *Npc1^−/−^* mouse. This study provides a comprehensive understanding of the STING interactomes and its regulatory network, shedding light on potential therapeutic targets for neurological diseases.

